# Editorial: Covid-19 and obesity

**DOI:** 10.3389/fendo.2022.1122274

**Published:** 2022-12-23

**Authors:** Valeria Guglielmi, Marwan El Ghoch, Silvia Bettini, Jeff M. P. Holly

**Affiliations:** ^1^ Department of Systems Medicine, University of Rome Tor Vergata, Rome, Italy; ^2^ Internal Medicine Unit - Obesity Center, University Hospital Policlinico Tor Vergata, Rome, Italy; ^3^ Department of Nutrition and Dietetics, Faculty of Health Sciences, Beirut Arab University, Beirut, Lebanon; ^4^ Center for the Study and the Integrated Treatment of Obesity, Internal Medicine 3, Padua University Hospital, Padua, Italy; ^5^ Faculty of Medicine, School of Translational Health Science, Bristol Medical School, University of Bristol, Southmead Hospital, Bristol, United Kingdom

**Keywords:** COVID-19, SARS-CoV-2, obesity, adipose tissue, pandemic (COVID-19), vaccine

The COVID-19 pandemic is reaching its third year and at the time of writing, approximately 641,900,000 people have been infected and over 6,622,000 deaths have been registered.

Importantly, approximately 50% of deaths related to COVID-19 have been in people with co-existing vascular and metabolic disorders (1). Among these, in addition to advancing age, a significant contributor to poorer outcomes is the coexistence of the SARS-CoV-2 infection with obesity.

Thanks to the development of vaccines and improved therapeutic approaches, daily global deaths have been markedly reduced. However, further pandemic spikes may be expected as virus mutation occurs, as shown by the rapid spread of the more recent SARS-CoV-2 variants, the waning of vaccine effectiveness, vaccination hesitancy and impaired immune responses. From this perspective, it remains crucial to continue to identify and understand the susceptibility of at-risk populations.

The present Research Topic, including eight review articles (three systematic reviews and metanalyses), five original papers, one brief research report and one opinion article, revisits some of the most important aspects of COVID-19 in people living with obesity, and summarizes the main insights into the field, collected during the early and more aggressive phases of the pandemic.

By analysing data relating to the hospitalizations of 176,137 patients throughout Germany, with a confirmed COVID-19 infection in 2020, Keller et al. found that patients with obesity were at increased risk of major adverse cardio- and cerebrovascular events, acute respiratory distress syndrome (ARDS), venous thromboembolism, intensive care unit (ICU) admission, mechanical ventilation and extracorporeal membrane oxygenation.

Considering the data of over 1,000 individuals from the UK Biobank, Morys and Dagher investigated whether metabolic health, defined by waist circumference, dyslipidaemia, hypertension, type 2 diabetes and systemic inflammation, on average 11 years prior to 2020, were related to increased rates of SARS-CoV-2 infection and mortality during the first phase of the COVID-19 outbreak. After controlling for confounding variables (i.e., socioeconomic status, age, sex and ethnicity), poor metabolic health resulted in a higher COVID-19 mortality but did not affect the risk of SARS-CoV-2 test positivity. Although all of these co-existing chronic conditions have been proven to be significant predictors of adverse outcomes in COVID-19 patients (2), Yu et al. provided evidence from the ORCHID study that obesity was independently linked to prolonged hospital stays in 116 COVID-19 patients without comorbidities.

Although many studies confirmed the positive association between the severity of COVID-19 and BMI (3, 4), a more in-depth approach has recently been taken to understanding the interactions between obesity and poor outcomes, following a SARS-CoV-2 infection. In particular, the importance of adipose distribution has been pointed out, with visceral adiposity being shown to be more predictive of a poorer outcome than subcutaneous fat. As a consequence, questions have been raised regarding the assessment of obesity *via* BMI, which can overlook the role of fat distribution and sarcopenia, potentially further increasing the risk of critical illness, especially among the elderly (Azzolino and Cesari). To disentangle the independent causal relationships of body fat mass and fat-free mass in relation to COVID-19 severity, Yoshiji et al. conducted a Mendelian randomization study using single nucleotide polymorphisms associated with body fat mass and fat-free mass in individuals of European ancestry from the UK Biobank, and analysed their effects on severe COVID-19 from the COVID-19 Host Genetics Initiative. In this analysis, only body fat mass was independently associated with severe COVID-19, indicating that the causal relationship between COVID-19 severity and obesity is likely to be mediated by adiposity.

The contribution of obesity to the severity of COVID-19 may be explained in multiple ways (5, 6).

Increasing evidence indicates that obesity could result in altered lung physiology, including reduced lung volumes, ventilation–perfusion abnormalities and respiratory muscle inefficiency, as well as management difficulties in critical care settings. Bhattacharya et al. also considered the many lessons learnt from the 2009 H1N1 influenza A pandemic, and discussed how increased inflammation and activation of the renin-angiotensin-aldosterone system (RAAS) may be factors contributing to COVID-19 severity, resulting in a further deterioration of the cardiovascular and lung functions of individuals with obesity. In addition, those with obesity present a hypercoagulability state that may potentiate COVID-19 coagulopathy, implicated in severe COVID-19 cases. Such a pro-thrombotic state is also promoted by the dysregulated immune responses observed in obesity and, in particular, orchestrated by inflammation, hypoxia and endothelial hyper-activation, as detailed by Gammone and D’Orazio.

This impaired antiviral immunity renders subjects with obesity more susceptible to the SARS-CoV-2 infection and disease progression (7), which are also likely to be due to higher levels of ACE2, the main receptor to SARS-CoV-2 entry into the host cell. Indeed, ACE2 has also been identified in adipocytes, enabling adipose tissue to serve as a functional viral reservoir especially in conditions of adiposity excess (Pasquarelli-do-Nascimento et al.). Thus, an accumulation of cardiac and perivascular adipose tissue may potentially act as a viral reservoir in heart proximities, locally mediating the detrimental effects of obesity. In obesity, even the reduced levels of Angiotensin (1–7), a vasoactive peptide generated by the enzymatic activity of ACE2 (directly from AngI and indirectly, *via* the activity of ACE, from AngII) with vasodilatory and cardioprotective effects, may contribute to the cardiac and haemodynamic complications of COVID-19 (Pasquarelli-do-Nascimento et al.). Finally, the so-called fat embolism syndrome was suggested as another mechanism expanding the major risk of severe disease in patients with obesity (8).

Despite the clear association between obesity and disease severity, many studies, such as those included in the metanalyses of Zhao et al. and Helvaci et al. Failed to confirm a significant impact of obesity on mortality (Peres et al.). Conversely, the metanalysis of Singh et al. which included 167 studies and over three million patients, identified a clear association between obesity and increased COVID-19 mortality. The meta-regression analysis indicated that half of the heterogeneity in mortality data could be explained by age, gender, diabetes, hypertension, pulmonary and cardiovascular diseases possibly accounting for these inconsistencies, together with differences in study populations, healthcare systems and threshold values for BMI.

Fortunately, a significant proportion of morbidity and mortality has been avoided by putting into action prevention strategies for patients with COVID-19 at risk of severe disease, including neutralizing monoclonal antibodies targeting SARS-CoV-2, novel oral antiviral agents and, above all, vaccines with high efficacy and levels of safety.

However, data from previous vaccine trials have shown defective immune responses to vaccinations against different viruses in people with obesity, therefore, attention has been raised regarding the reduced vaccine-induced immunity in such patients. Data from the phase III SARS-CoV-2 vaccine trials on Pfizer, Moderna and Johnson & Johnson formulations indicated a similar efficacy in individuals with and without obesity. However, these outcomes were not statistically validated, and subsequent clinical trials have reported decreased antibody titers and weakened immune responses following the SARS-CoV-2 vaccination linked to obesity; therefore, the effectiveness and durability of these vaccines in individuals with obesity are still matters of debate (Nasr et al.)

If the presence of obesity is detrimental to the COVID-19 outcome, on the other hand, the COVID-19 pandemic has made the treatment of obesity even more challenging. Indeed, the lockdown conditions, which resulted in a decline in eating habits and psychological well-being, sleep disruption and mobility restrictions, as well as the deferral of bariatric surgery interventions have, in turn, worsened the obesity epidemic (9). Indeed, Yang et al. who examined the changes in obesity, physical activity and nutrient intake during the COVID-19 epidemic in 2020, using the Korean National Health and Nutritional Examination Survey (KNHANES) database, found that the obesity rate in Korea significantly increased by comparison with the expected obesity rate of 2019, especially among men, mainly due to a decrease in physical activity.

In this context, to counteract the detrimental effects of the pandemic on obesity prevalence, the effectiveness of self-managed weight loss through the use of smart body fat scales has been investigated by Huang et al. in over 100,000 Chinese adult users registered in 2020. The authors found that many participants with overweight/obesity achieved weight loss goals by smart body fat scales, and the effectiveness of weight and fat loss was greater among participants with obesity rather than those with overweight.

In conclusion, obesity is now well accepted as a substantial risk factor for poor outcomes following the SARS-CoV-2 infection, therefore, given the ongoing nature of the pandemic, constant attention and proactive clinical management are needed for patients with obesity in order to reduce morbidity and mortality from SARS-CoV-2. This also adds further motivation to the ongoing efforts to curb the prevalence of obesity, in addition to the many other recognised health benefits of maintaining an appropriate weight.

## Author contributions

All authors listed have made a substantial, direct and intellectual contribution to the work, and have approved it for publication.
